# Transition Metal(II) Complexes with Cefotaxime-Derived Schiff Base: Synthesis, Characterization, and Antimicrobial Studies

**DOI:** 10.1155/2014/926287

**Published:** 2014-02-12

**Authors:** Aurora Reiss, Mariana Carmen Chifiriuc, Emilia Amzoiu, Cezar Ionuţ Spînu

**Affiliations:** ^1^Department of Chemistry, Faculty of Mathematics and Natural Sciences, University of Craiova, 107I Calea Bucureşti, 200478 Craiova, Romania; ^2^Department of Microbiology, Faculty of Biology, University of Bucharest, 1-4 Aleea Portocalelor, 60101 Bucharest, Romania; ^3^Faculty of Pharmacy, University of Medicine and Pharmacy of Craiova, 66, 1 May Street, 200638 Craiova, Romania

## Abstract

New [ML_2_(H_2_O)_2_] complexes, where M = Co(II), Ni(II), Cu(II), and Zn(II) while L corresponds to the Schiff base ligand, were synthesized by condensation of cefotaxime with salicylaldehyde *in situ* in the presence of divalent metal salts in ethanolic medium. The complexes were characterized by elemental analyses, conductance, and magnetic measurements, as well as by IR and UV-Vis spectroscopy. The low values of the molar conductance indicate nonelectrolyte type of complexes. Based on spectral data and magnetic moments, an octahedral geometry may be proposed for Co(II), Ni(II), and Zn(II) complexes while a tetragonal geometry for Cu(II) complex. Molecular structure of the Schiff base ligand and its complexes were studied using programs dedicated to chemical modeling and quantomolecular calculation of chemical properties. All the synthesized complexes were tested for *in vitro* antibacterial activity against some pathogenic bacterial strains, namely *Escherichia coli, Klebsiella pneumoniae, Pseudomonas aeruginosa, Bacillus subtilis,* and *Staphylococcus aureus*. The MIC values shown by the complexes against these bacterial strains revealed that the metal complexes possess superior antibacterial activity than the Schiff base.

## 1. Introduction

Infectious diseases caused by bacteria remain a major worldwide health problem due to rapid development of resistance to the existing antimicrobial drugs. Considering the emergence of pathogenic bacteria multidrug resistant strains, the discovery of new antimicrobial compounds is highly important. Cephalosporins (also named *β*-lactams) are synthetic antibiotics active against Gram-negative and Gram-positive bacteria by inhibiting the synthesis of the peptidoglycan layer from the cell wall [1]. The antibiotics of this class have in their structure a *β*-lactam ring that helps the antibiotic to bind to the enzymes that synthesize the peptidoglycan layer and perturbs the process. The mechanism that makes bacteria resistant to *β*-lactams is due to the synthesis of *β*-lactamase enzymes that break the *β*-lactam ring and the antibiotic cannot bind to peptidoglycan layer [2].

The antibiotic cefotaxime is the third generation cephalosporins which possess increased activity against bacteria than the first and second generation cephalosporins. Many drugs possess better pharmacological properties when they are in the form of metal complexes. So, the literature presents many metal complexes of different cephalosporins and their biological activity [3–16], but only a few studies present metal complexes with Schiff base derived from cephalosporins [17–22].

In this context, the aim of this study was to assess the antimicrobial activity of some newly synthesized compounds of transition metallic ions Co(II), Ni(II), Cu(II), and Zn(II) with the Schiff base derived from cefotaxime with salicylaldehyde.

## 2. Experimental

### 2.1. Materials

All the chemicals (E. Merk, Germany) were used without further purification. All metal salts used were chloride (CuCl_2_·2H_2_O, NiCl_2_·6H_2_O, CoCl_2_·6H_2_O, or ZnCl_2_·2H_2_O). Solvents used were of analytical grade.

### 2.2. Synthesis of the Schiff Base

A solution of cefotaxime sodium (1 mmol in 40 mL methanol) was added drop-wise, under stirring to a solution of salicylaldehyde (1 mmol in 10 mL methanol). A solution of 0.1 M NaOH was added to adjust the pH at 7-8 and the reaction mixture was refluxed for 3 h. The resulting product was filtered off, washed with distilled water and methanol, and dried under vacuum. Recrystallization from a mixture of ethanol-propanol (50 : 50) gave the Schiff base. M.p. 150–153°C (found: C, 47.35; H, 3.41; N, 12.00% Calcd. for C_23_H_20_S_2_O_8_N_5_Na: C, 47.50; H, 3.44; N, 12.04%).

### 2.3. Syntheses of the Metal Complexes

The metal complexes were prepared following the same method. The Schiff base was prepared *in situ* from the precursors as follows: a solution of cefotaxime sodium (2 mmol in 40 mL ethanol) was added to a solution of salicylaldehyde (2 mmol in 10 mL ethanol). The resulting mixture was refluxed for 3 h and then a methanolic solution 0.1 M of NaOH was added. In the next step, the metal salts (1 mmol in 15 mL ethanol) were added to the ligand solution under continuous stirring when the complex was precipitate. The obtained colored product was separated by filtration, washed with distilled water and methanol, and dried under vacuum. Recrystallization from hot methanol gave the metal complexes:

 CoL_2_(H_2_O)_2_ (found: C, 43.78; H, 3.33; N, 11.13; M, 4.79%);
 Calcd.: CoC_46_H_42_S_4_O_18_N_10_Na_2_: C, 43.98; H, 3.34; N, 11.15; M, 4.69%;
 NiL_2_(H_2_O)_2_: found: C, 43.89; H, 3.33; N, 12.13; M, 4.58%;
 Calcd.: NiC_46_H_42_S_4_O_18_N_10_Na_2_: C, 43.99; H, 3.34; N, 11.15; M, 4.68%;
 CuL_2_(H_2_O)_2_ (found: C, 43.80; H, 3.32; N, 11.10; M, 5.03%);
 Calcd.: CuC_46_H_42_S_4_O_18_N_10_Na_2_: C, 43.82; H, 3.33; N, 11.12; M, 5.04%;
 ZnL_2_(H_2_O)_2_ (found: C, 43.56; H, 3.33; N, 11.07; M, 5.15%);
 Calcd.: ZnC_46_H_42_S_4_O_18_N_10_Na_2_: C, 43.76; H, 3.33; N, 11.09; M, 5.17%.

### 2.4. Analyses and Instrumentation

The IR spectra were recorded on a Perkin Elmer 157 instrument in anhydrous KBr pellets in the range of 4000–400 cm^−1^. A Unicam UV2-300 spectrometer was used to obtain electronic spectra in DMF solutions. The molar conductivities were determined by using OK-102 (Hungary) conductivity meter. The melting points were determined by using Sanyo Gallenkamp apparatus. The magnetic susceptibility measurement was made on a Faraday balance at room temperature. The metal contents of the complexes were determined by atomic absorption technique using Varian-AA775 spectrophotometer. C, H, and N were analysed using M.L.W. microelementary CHN analyser. The heating curves (TG and DTA) were carried out in a Netzsch TG 209C thermobalance with a sample mass of 10 mg over the temperature range of 20–1000°C using a heating rate of 10°C. The measurements were carried out in nitrogen atmosphere (flow rate of 40 mL min^−1^) using alumina crucibles. The ^1^H and ^13^C NMR spectra were recorded on a Varian Gemini 300BB at room temperature in the same solvent CDCl_3_.

### 2.5. Antimicrobial Assay

#### 2.5.1. Antimicrobial Activity

*In vitro *antimicrobial tests were carried out by an adapted agar-disc diffusion technique using 0.5 McFarland suspension of bacteria obtained from 24 h cultures. The antimicrobial activities of the synthesized compounds were determined against six Gram-negative (*Escherichia coli *13529*, Escherichia coli *12147, *Klebsiella pneumoniae *1204*, Klebsiella pneumoniae *13420, *Pseudomonas aeruginosa *1246, and *Pseudomonas aeruginosa *13202) microbial strains and three Gram-positive (*Staphylococcus aureus *MRSA 1263*, Staphylococcus aureus *13209, and *Bacillus subtilis *12488) microbial strains. The compounds were solubilised in dimethylsulfoxide to a final concentration of 1 mg/mL. A volume of 10 *μ*L of each tested compounds solution was distributed directly on the solid medium previously seeded with the microbial inoculums. The inoculated plates were incubated for 24 h at 37°C. Antimicrobial activity was assessed by measuring the growth inhibition zones diameters [23–27].

#### 2.5.2. Determination of Minimum Inhibitory Concentration (MIC)

The quantitative assay of the minimal inhibitory concentration (MIC, *μ*g/mL) was based on liquid medium twofold microdilutions. After 24 h of incubation at 37°C, the bactericidal activity was quantified by measuring the absorbance of the liquid culture at 620 nm.

#### 2.5.3. Assessment of Biofilm Development on the Inert Substratum by the Micro-Titer Method

At the end of the MIC assay experiment, the plastic wells were emptied, washed three times with phosphate buffered saline (PBS), and fixed with cold methanol and the bacterial biofilm formed on the plastic walls was stained with 1% violet crystal solution for 30 minutes and then resuspended in 30% acetic acid. The intensity of the colored suspensions was assessed by measuring the absorbance at 490 nm; the obtained values being directly proportional with the number of bacterial cells adhered to the plastic wall [23–27].

## 3. Results and Discussion

The Schiff base ligand was prepared by refluxing the appropriate amount of cefotaxime with salicylaldehyde in methanol. The structure of synthesized Schiff base ligand was established by ^1^H and ^13^C NMR spectra, IR spectrum and microanalytical data. The metal complexes of ligand were prepared by the stoichiometric reaction of the corresponding metal(II) chloride with the ligand in a molar ratio M : L of 1 : 2. The complexes were obtained as air-stable amorphous solids which decompose without melting. They are insoluble in water, partially soluble in methanol and ethanol, totally soluble in DMF and DMSO. The molar conductivities of the complexes measured in DMF (10^-3 ^mol·L^−1^ at room temperature) have lower values (13–18 Ω^−1^ cm^2^  mol^−1^) indicating their nonelectrolyte nature [28]. The elemental analysis and the physical measurements permit the suggestion of the empirical formulae: ML_2_(H_2_O)_2_ where L = Schiff base and M = Co(II), Ni(II), Cu(II) and Zn(II).

### 3.1. ^1^H and ^13^C NMR Spectra

**Figure 1:**
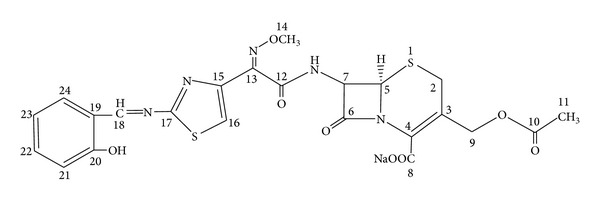
Chemical structure of the Schiff base.

The ^1^H and ^13^C NMR spectra of Schiff base and diamagnetic Zn(II) complex were performed. In the ^1^H NMR spectrum of the ligand, the formation of Schiff base is supported by the presence of a singlet at *δ* = 8.7 ppm corresponding to the azomethine proton (–N=CH–) and a peak at *δ* = 163.62 ppm in the ^13^C-NMR spectrum (Table 1) [29]. The chemical structure of the Schiff base is presented in Figure 1.

The signal assigned to the azomethine proton in ^1^H NMR spectrum of Schiff base shifted downfield in the spectrum of the Zn(II) complex (*δ* = 8.96 ppm) indicating coordination through the azomethine nitrogen to the metallic ion. The signal of the phenolic proton in the free ligand at *δ* 12.8 ppm (intramolecularly H-bonded phenolic group) is absent in the spectrum of the complex, thus confirming the deprotonation of the phenolic group on complex formation.

The signal of the carbon atom from the azomethine group in ^13^C-NMR spectrum of the ligand is slightly shifted downfield (*δ* = 168.16 ppm) in the spectrum of the Zn(II) complex due coordination.

### 3.2. Infrared Spectra

The IR data of the free cephalosporin, the Schiff base ligand (L), and its complexes are presented in Table 2.

A comparison of the IR spectra of the free cephalosporin and the Schiff base ligand gives us the proof about the formation of the ligand Schiff base (L) between the cefotaxime-Na and salicylaldehyde. The main bands in the IR spectrum of the free cephalosporin (cefotaxime-Na) are at 3442, 1776, and 1640 cm^−1^ attributed to *ν*(NH_2_), *ν*(C=O)*β*-lactam, and *ν*(C=O)amide + *ν*(C=O)ester, respectively. The IR Schiff base spectrum shows absorption bands at 2800, 1657, and 1274 cm^−1^. The broad absorption band at *ca.* 2800 cm^−1^ is due to an intramolecular hydrogen bond which shows the presence of –OH group in the ligand molecule which is situated in a favourable position (orto) towards the azomethine group to form this type of bond. The absorption band at 1657 cm^−1^ is attributed to *ν*(C=N) stretching vibration and the absorption band 1275 cm^−1^ is attributed to *ν*(C–O) phenolic. The bands attributed to *ν*(C=O)*β*-lactam and *ν*(C=O)amide + *ν*(C=O)ester which are also presented in the IR spectrum of Schiff base are slightly shifted at 1770 and 1645 cm^−1^, respectively.

**Figure 2:**
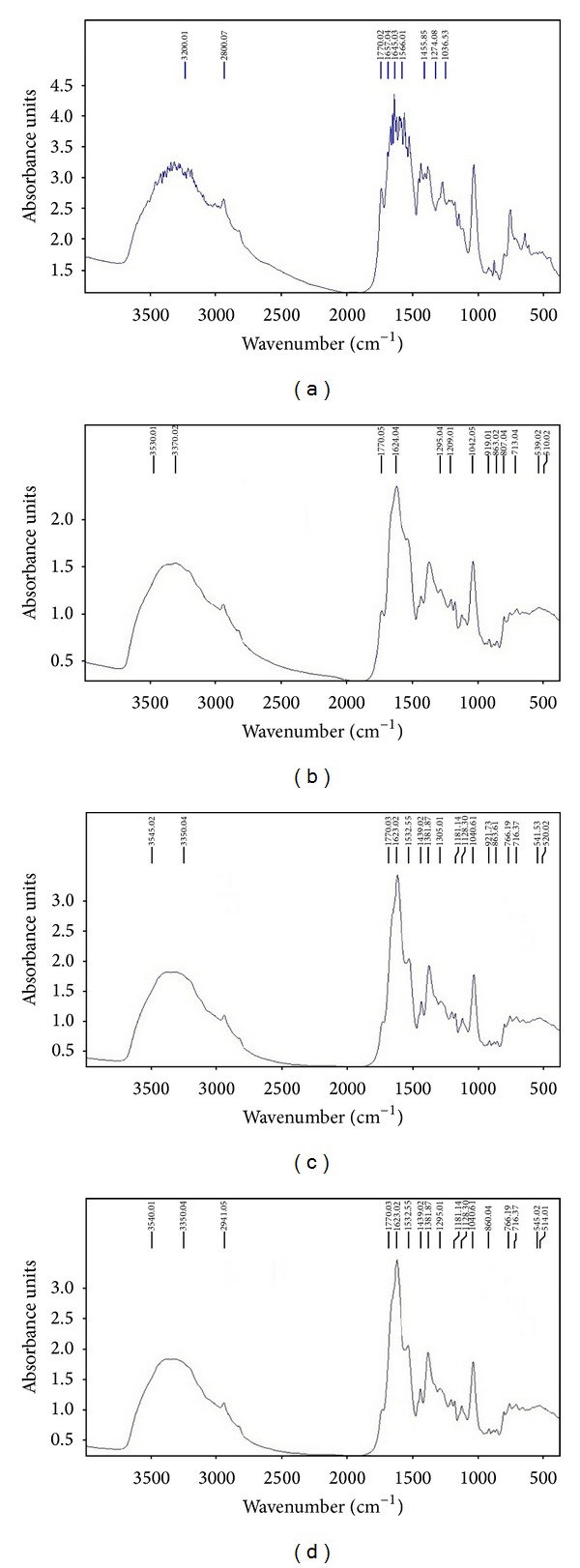
IR spectra of the Schiff base (a) and its complexes: [CoL_2_(H_2_O)_2_] (b); [NiL_2_(H_2_O)_2_] (c); [CuL_2_(H_2_O)_2_] (d).

A comparison of the IR spectra of the complexes and those of the free Schiff base ligand allows us to determine the coordination sites that could be involved in chelation process (Figure 2). The spectra of these complexes contain a broad band around 3530–3545 cm^−1^ attributed to *ν*(OH). Additionally, the coordinated water presents *δ*r(H_2_O) rocking at 857–863 cm^−1^ and *δ*w(H_2_O) wagging at 539–545 cm^−1^ [30, 31]. The band at 1657 cm^−1^ attributed to *ν*(HC=N) from the Schiff base is shifted to lower values (1620–1630 cm^−1^) in the complexes, which suggests that the Schiff base ligand is coordinated to the metallic ion by the N atom in the azomethine group. The absorption band at 2800 cm^−1^ attributed to the formation of a intramolecular hydrogen bonding does no longer appear in the complexes spectra, which proves the deprotonation of –OH group. Likewise, the absorption band at 1274 cm^−1^ in the ligand spectrum attributed to the *ν*(C–O) phenolic frequency appears at ~21–36 cm^−1^ higher frequencies. These shifts indicate the participation of the O atom of the deprotonated hydroxyl group in the formation of the M–O bonds. The metal complexes are also characterized by the appearance of some new bands at 510–520 cm^−1^ and 419–423 cm^−1^, which are assigned to *ν*(M–O) and *ν*(M–N) stretching frequencies, respectively. The *ν*(C=O)*β*-lactam and *ν*(C=O)amide + *ν*(C=O)ester frequencies at 1770 cm^−1^ and 1640 cm^−1^, respectively, in the Schiff base spectrum are not shifted in the complexes spectra, which means that these groups are not involved in the coordination. In conclusion, we can say that the Schiff base ligand is bidentately coordinated to the metallic ions with N and O atoms from azomethine and phenolic groups.

### 3.3. Electronic Spectra and Magnetic Moment Values

**Figure 3:**
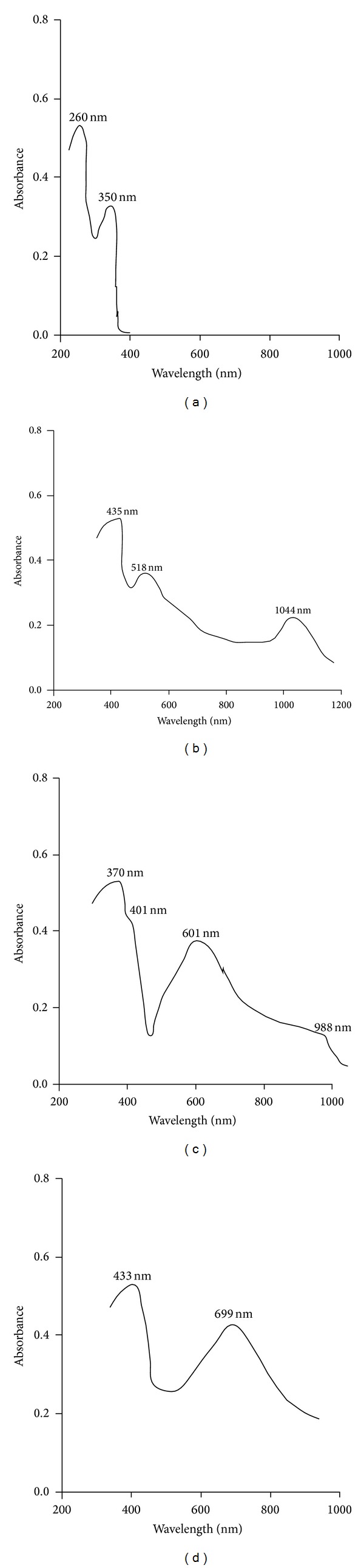
UV-Vis spectra of the Schiff base (a) and its complexes: [CoL_2_(H_2_O)_2_] (b); [NiL_2_(H_2_O)_2_] (c); [CuL_2_(H_2_O)_2_] (d).

In order to obtain information regarding the coordination geometry of the complexes, the electronic spectra were determined at room temperature in DMF (Figure 3) and the data obtained were correlated with magnetic moment values and ligand field parameters: splitting energy (10Dq), interelectronic repulsion parameter (*B*), and nephelauxetic ratio (*β*) (Table 3).

The electronic spectra of the ligand present two absorption bands at 38460 and 28570 cm^−1^, respectively, attributed to *π* → π* and *n* → π*, respectively, determined by the C=O and C=N groups. These absorption bands also appear in the electronic spectra of the complexes, but they are shifted to ~1500–5000 cm^−1^ lower values, which proves the coordination of the ligand to the central metallic ions. The electronic spectra of the Co(II) complex display two bands at 9570 cm^−1^ (*ν*_1_) and 19305 cm^−1^ (*ν*_3_), which are assigned to^4^T_1 g_ → ^4^T_2 g_ (F)(*ν*_1_) and ^4^T_1 g_ (F) → ^4^T_1 g_ (P)(*ν*_3_) transitions, respectively. These are the characteristic bands of high spin octahedral Co(II) complexes [32]. The ligand field parameters (Dq, *B*, and *β*) are calculated using E. Koning equations [33], when only *ν*_3_ and *ν*_1_ bands are observed in the electronic spectra and the values are well within the range reported for the octahedral complexes [32, 34]. The value of the magnetic moment is 4.78 BM for Co(II) complex which suggests three unpaired electrons in an octahedral environment [35]. The electronic spectrum of Ni(II) complex presents three d → d absorption bands at 10120, 16630, and 24880 cm^−1^ in octahedral environment corresponding to ^3^A_2 g_ → ^3^T_2 g_(F)(*ν*_1_), ^3^A_2 g_ → ^3^T_1 g_ (F)(*ν*_2_), and ^3^A_2 g_ (F) → ^3^T_1 g_ (P)(*ν*_3_) transitions [32, 34]. The values obtained for Dq, *B*, and *β* are in agreement with the experimental ones for Ni(II) octahedral complexes [33]. The magnetic moment value of Ni(II) complex is 3.12 BM indicating the presence of two unpaired electrons on Ni(II) ion and suggesting this complex to have an octahedral geometry [35, 36]. The electronic spectrum of Cu(II) complex presents only one broad band with maximum centered at 14290 cm^−1^ typical for the copper (II) ion in an elongated distorted octahedral (tetragonal) geometry [32, 34]. The magnetic moment value of Cu(II) complex is 1.86 BM which indicates the presence of one unpaired electron on Cu(II) ion in a d^9^ system [37]. The electronic spectrum of Zn(II) complex does not contain d → d transitions, but presents only one band at 19800 cm^−1^, which may be attributed to a L → M charge transfer [32]. The Zn(II) complex was found to be diamagnetic as expected.

### 3.4. Thermal Studies

**Figure 4:**
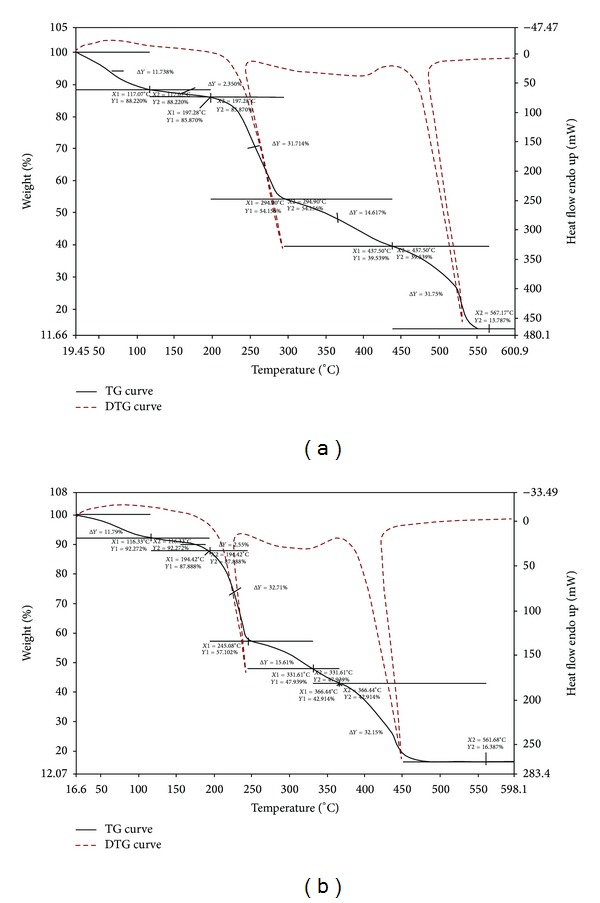
Thermogravimetric curves for Ni(II) complex (a) and Cu(II) complex (b).

**Scheme 1:**
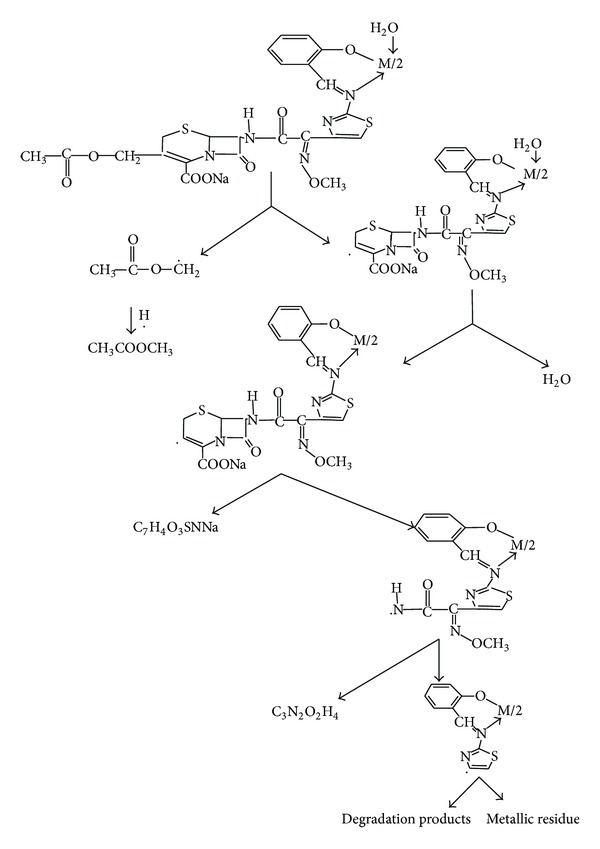
Fragmentation of Co(II), Ni(II), and Cu(II) complexes following the thermal decomposition.

Thermogravimetric analyses for the Co(II), Ni(II), and Cu(II) complexes were carried out from room temperature to 900°C and show nearly the same pattern (Figure 4). Calculated and experimental mass losses are comparable. The data are given in Table 4. At 70–127°C, the experimental mass loss of 11.53–11.79% may be due to the loss of two CH_3_COOCH_3_ molecules in complexes. Weight loss in the range 117–207°C with experimental mass loss of 2.35–2.55% in all the complexes indicates the loss of two coordinated water molecules (calculated value, 2.86%). This temperature required for water loss indicates that water molecules are strongly bonded to the metal ion and this type of thermal behaviour is characteristic of coordinated water molecules [38]. From 197°C to 463°C, a sharp decrease in weight indicated the loss of fragments from two Schiff base molecules from the complexes with experimental mass loss of 46.32–48.32% for all the complexes, respectively. In the final stage, which occurs in the 453–605°C temperature range, both decomposition products with experimental mass loss of 31.75–32.75% for the three complexes and a black residue are eliminated. Chemical analysis of the black final residue corresponds to the metallic oxide. The stages of thermal decomposition for the Co(II), Ni(II), and Cu(II) complexes may be summarized by the scheme presented in Scheme 1. In conclusion, TG experiments revealed the nature of complex species as anhydrous and confirmed their compositions suggested by the analytical data.

### 3.5. Molecular Modelling

Molecular structure of the Schiff base ligand and its metal complexes were studied using programs dedicated to the chemical modeling and quantomolecular calculations of chemical properties. The molecular geometries of the compounds were obtained by molecular orbitals quantum methods, with the optimization being done using the molecular mechanics program Hyperchem 8 [39]. Some of the structural data (called descriptors) obtained by Hyperchem program are presented in Table 5.

**Figure 5:**
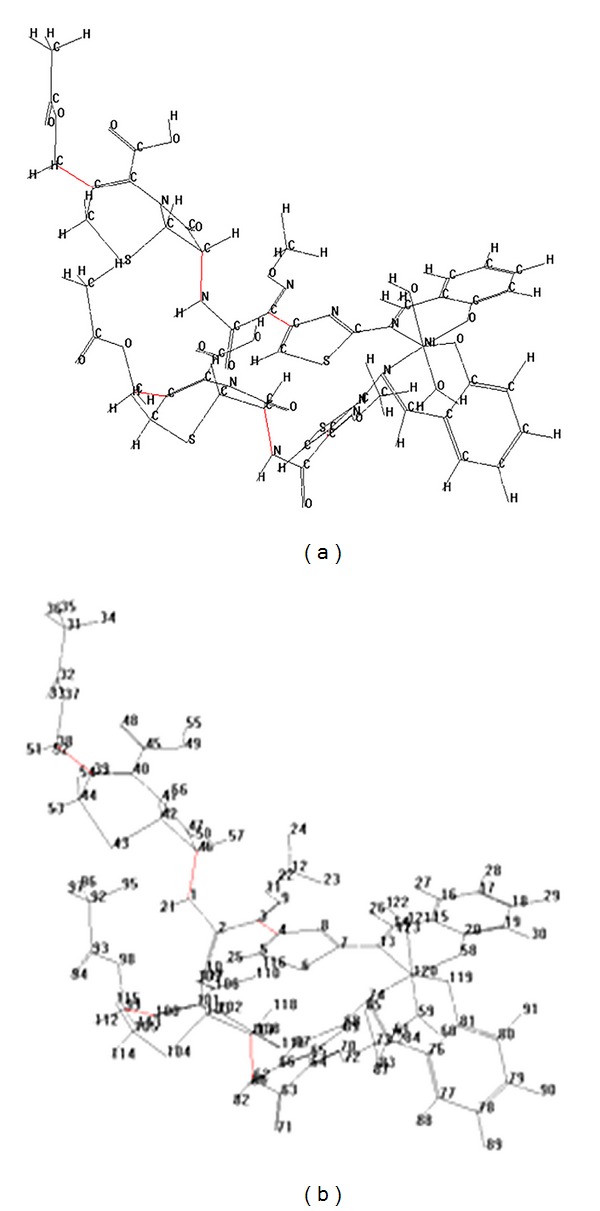
Molecular structure of the complex [NiL_2_(H_2_O)_2_].

In Figure 5 the molecular structure of the complex [NiL_2_(H_2_O)_2_] is presented, in which the interatomic bonds in red correspond to the labile areas of the molecule, areas where the interatomic bonds break after thermal degradation. This fact is confirmed by the increase in the interatomic distances in the complex compared with the ligand: distance C_38_–C_39_ (from 1.5182 Å in the ligand to 1.5297 Å in the complex), distance N_1_–C_46_ (from 1.4435 Å in the ligand to 1.4520 Å in the complex), and distance C_3_–C_4_ (from 1.3486 Å in the ligand to 1.3596 Å in the complex), respectively. Similar structural changes are also found in the other complexes. Instead, the interatomic distances that decrease in length after coordination indicate an increase in their stability. All these results are consistent with the results of the thermogravimetric analysis.

The structural analysis of the chemical compounds included in this study led to other parameters, *E*: total energy of the molecule, *M*: molecular mass, *V*: molecular volume, *α*: molecular polarizability; *μ*: dipole moment, and OV: ovality index, which are presented in Table 6.

The results presented in Table 6 indicate the following.

- Total energy *E* has the highest value (287.481 kcal/mol) for the complex [CuL_2_(H_2_O)_2_], which explains its high reactivity and the lowest value (277.223 kcal/mol) for the complex [NiL_2_(H_2_O)_2_], which explains its increased stability. These results are accordant with the biological activity which indicates that the lowest antimicrobial spectrum was noticed for the Ni(II) complex, while the largest inhibitions zones were exhibited by the Cu(II) complex (Table 7); this last complex compound proved to be the most efficient also in the quantitative assay (Table 8).
- The variation of the *V*, *μ*, and *α* descriptors, calculated and presented in Table 6, is consistent with the molecular mass of the complex compounds: the values of these descriptors increase along with the increase in the molecular mass.
- The values of the molecular shape descriptor OV (index oval), which expresses the ratio between the diameters of the smallest ellipsoid which contains a complex molecule, vary inversely proportionally to the molecular mass of the compounds.

**Figure 6:**
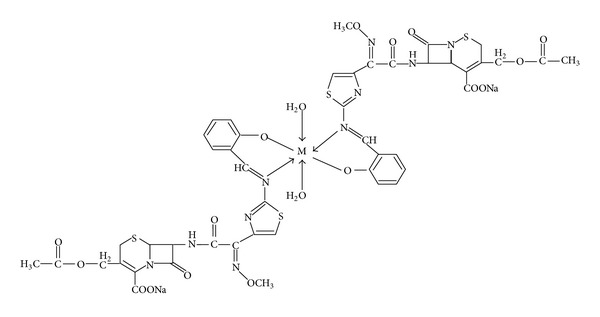
Proposed structures of the metal(II) complexes.

On the basis of the above data, the proposed structures for the complexes [ML_2_(H_2_O)_2_] where L = Schiff base and M = Co(II), Ni(II), Cu(II), and Zn(II) are shown in Figure 6.

### 3.6. *In Vitro* Antimicrobial Discussion

The qualitative screening of the susceptibility spectra of various microbial strains to newly synthesized compounds showed that all tested compounds exhibited antimicrobial effect quantified by the occurrence of a growth inhibition zone (Table 7). For all tested complexes, the diameters of the inhibition zones were superior to those exhibited by DMSO alone and, also, those exhibited by the ligand and cefotaxime, suggesting that the antimicrobial activity of the obtained complexes is clearly superior to that of the ligand. The lowest antimicrobial spectrum was noticed for the Ni(II) complex, while the largest inhibitions zones were exhibited by the Cu(II) complex. The largest antimicrobial spectrum was obtained for Co(II) complex, which proved to be active against all tested microbial strains.

The results from the quantitative assay revealed that the tested compounds had a low inhibitory activity on bacterial growth. The absorbance values measured at 620 nm of the bacteria grown in presence of the tested compounds were similar to those of the organic solvent used (DMSO) in the majority of cases, excepting Ni(II) complex for *E. coli, *Co(II) complex for *E. coli, K. pneumoniae, *and Cu(II) complex for *E. coli, K. pneumoniae *and *S. aureus*, this last complex proving to be the most efficient also in the quantitative assay (Table 8).

**Figure 7:**
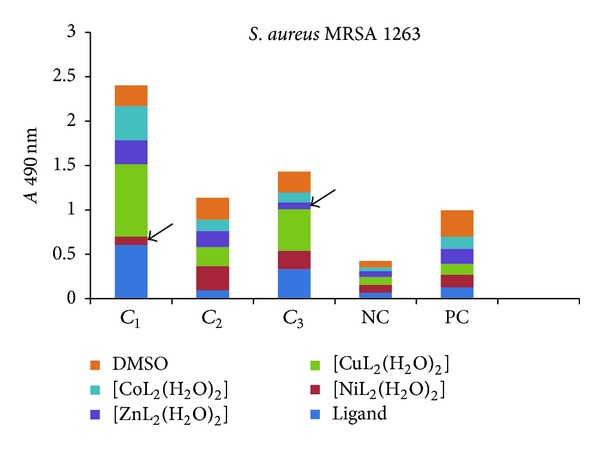
The intensity of *S. aureus* 1263 biofilm (quantified by *A* 490 nm on *y*-axis) developed in the presence of three successive binary concentrations, that is, 1 mg/mL, 500 *μ*g/mL, and 250 *μ*g/mL of the tested compounds (NC: negative, sterility control; PC: positive, growth control).

**Figure 8:**
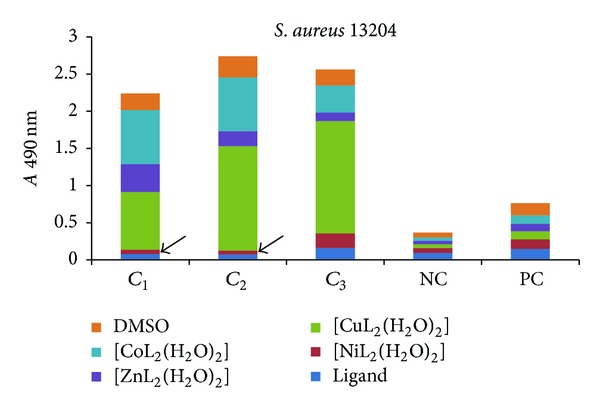
The intensity of *S. aureus* 13204 biofilm (quantified by *A* 490 nm on *y*-axis) developed in the presence of three successive binary concentrations, that is, 1 mg/mL, 500 *μ*g/mL and 250 *μ*g/mL of the tested compounds (NC: negative, sterility control; PC: positive, growth control).

**Figure 9:**
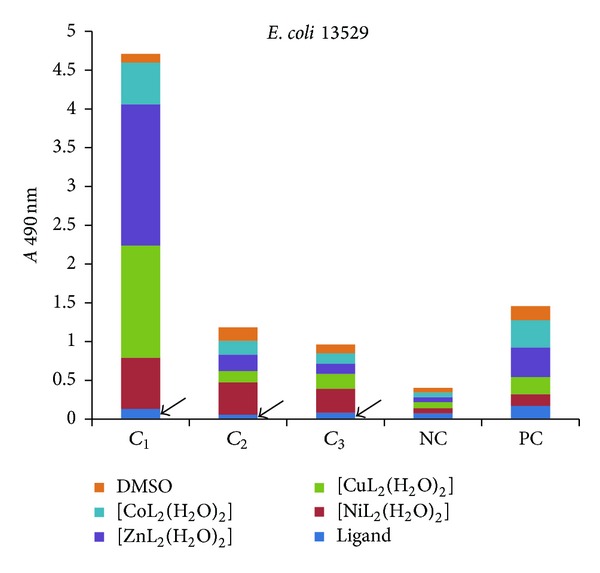
The intensity of *E. coli *13529 biofilm (quantified by *A* 490 nm on *y*-axis) developed in the presence of three successive binary concentrations, that is, 1 mg/mL, 500 *μ*g/mL, and 250 *μ*g/mL of the tested compounds (NC: negative, sterility control; PC: positive, growth control).

The influence of the tested compounds on the biofilm development on inert substrata was tested only on the microbial strains for which there have been registered good results in the previous assays, that is, *S. aureus* 1263, MRSA 13204, *E. coli* 13529, *E. coli *13147, *K. pneumoniae *13420, and *K. pneumoniae *1204. The obtained results showed that some of the compounds inhibited the ability of bacterial cells to colonize the inert substratum represented by the plastic wells, as revealed by the decreased values of *A* 490 nm, while some other compounds stimulated the biofilm development, as revealed by the increased values recorded for the *A* 490 nm, as compared with the positive control, represented by the microbial biofilm developed in the absence of the tested compounds (Figures 7–9).

Three successive concentrations of the obtained compounds, starting with *MIC*, that is, 1 mg/mL (noted *C*_1_), followed by two subinhibitory concentrations, that is, 500 *μ*g/mL (noted *C*_2_) and 250 *μ*g/mL (noted *C*_3_), were tested for their influence of the bacterial biofilm development. The antibiofilm activity of the tested compounds was generally lower than that exhibited by the ligand alone, excepting some few cases, detailed below.

In case of *S. aureus *strains, the complexes of Ni(II) (at concentrations ranging from 1 mg/mL to 500 *μ*g/mL), as well as of Zn(II) (at 250 *μ*g/mL), exhibited antibiofilm activity superior to that of the ligand (Figures 7 and 8).

**Figure 10:**
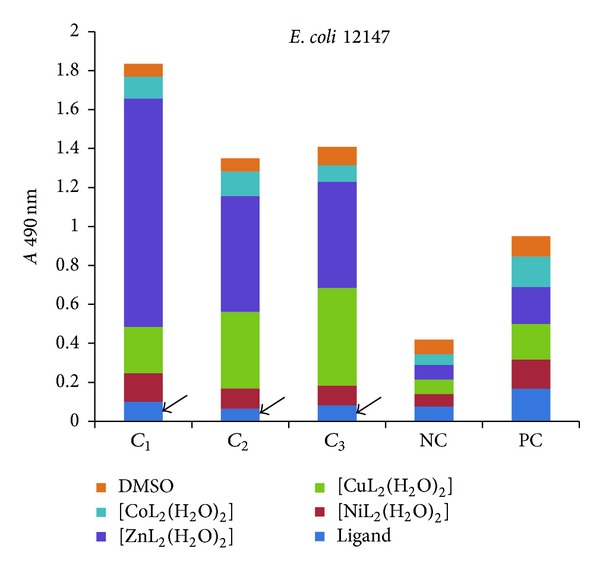
The intensity of *E. coli *12147 biofilm (quantified by *A* 490 nm on *y*-axis) developed in the presence of three successive binary concentrations, that is, 1 mg/mL, 500 *μ*g/mL, and 250 *μ*g/mL of the tested compounds (NC: negative, sterility control; PC: positive, growth control).

In case of *E. coli* strains, the biofilm development was significantly inhibited by all tested concentrations of the ligand (Figures 9 and 10).

**Figure 11:**
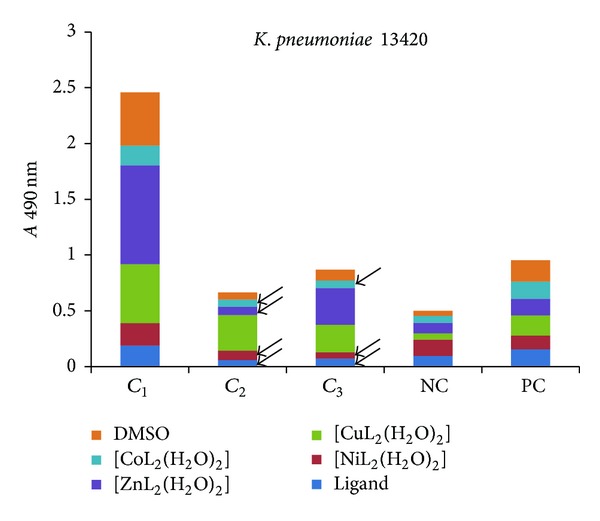
The intensity of *K. pneumoniae* 13420 biofilm (quantified by *A* 490 nm on *y*-axis) developed in the presence of three successive binary concentrations, that is, 1 mg/mL, 500 *μ*g/mL, and 250 *μ*g/mL of the tested compounds (NC: negative, sterility control; PC: positive, growth control).

**Figure 12:**
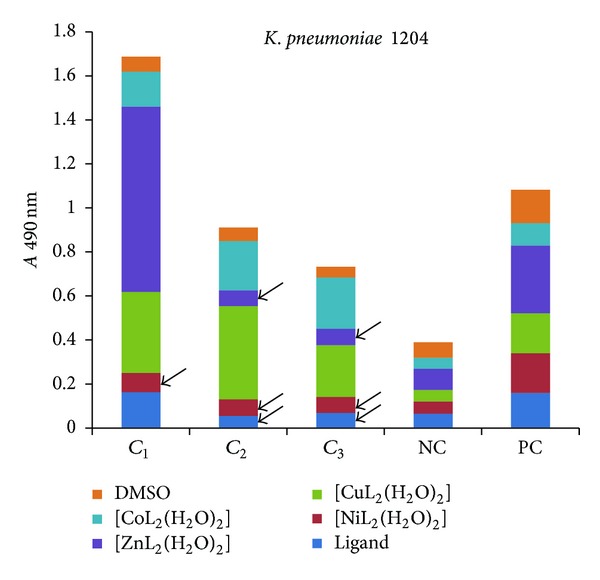
The intensity of *K. pneumoniae* 1204 biofilm (quantified by *A* 490 nm on *y*-axis) developed in the presence of three successive binary concentrations, that is, 1 mg/mL, 500 *μ*g/mL, and 250 *μ*g/mL of the tested compounds (NC: negative, sterility control; PC: positive, growth control).

In case of *K. pneumoniae* strains (Figures 11 and 12), the antibiofilm activity of the tested compounds varied with the tested concentration. At the MIC corresponding concentration (*C*_1_ of 1 mg/mL) only the complex of Ni exhibited anti-biofilm activity against one of the two tested strains (Figure 12). At the other two subinhibitory concentrations (*C*_2_ of 500 *μ*g/mL and *C*_3_ of 250 *μ*g/mL), the ligand, as well as the complexes of Ni(II), Zn(II) and Co(II) exhibited an anti-biofilm activity.

These results are consistent with the fact that the growth in biofilms is accompanied by an increased tolerance up to 10 to 100, or even more, 1000 to 4000 times higher than those established on planktonic/free cells, by the standard antibiotic susceptibility testing methods, evidently leading to clinical therapeutic failures of normal therapeutic doses of antibiotics. On the other side, the fact that, in some cases, the tested compounds exhibited antibiofilm activity at subinhibitory concentrations is suggesting their potential use as antibiofilm agents. The antibiofilm activity of the subinhibitory concentrations of the tested substances could be explained by the inhibition of bacterial metabolic pathways, implicated in the synthesis of different microbial components, including those implicated in different phases of the adherence and biofilm development processes. The fact that *K. pneumoniae* strains proved to be the most susceptible to the tested compounds concerning the ability to form biofilms on inert substrata could suggest that the tested compounds interfere with the synthesis of glycocalyx, the polysaccharidic substance which can form an organized structure called capsule, or an amorphous layer called slime, implicated in the formation of biofilms on inert surfaces.

Tweedy's chelation theory [40] offers an explanation for the increased antimicrobial activity of the metal complexes. In the chelated complex, the positive charge of the metal ion is partially shared with the donor atoms of the ligand and *π*-electron delocalization occurs over the whole chelate ring. In this way, the lipophilic character of the metal chelate is increasing and favouring its permeation through the lipoid layers of the bacterial membranes and blocking the metal binding sites in the microorganism.

## 4. Conclusions

Four metal(II) complexes with the Schiff base derived from cefotaxime with salicylaldehyde have been synthesized and characterised. Data from IR spectra concluded that the ligand behaves as a bidentate ligand coordinated in all the complexes. Electronic spectra and magnetic measurements indicate an octahedral geometry for Co(II), Ni(II), and Zn(II) complexes and a tetragonal geometry for Cu(II) complex. The results from the biological activity demonstrated that the newly synthesized complexes could exhibit, in some cases, improved antimicrobial activity against both planktonic and biofilm embedded cells, superior to that of the ligand and the included antibiotic, so, they could be used for the development of novel antimicrobial materials or strategies for fighting medical biofilms pathogens frequently implicated in the etiology of biofilm associated chronic infection.

## Figures and Tables

**Table 1:** ^
1^H and ^13^C NMR spectral data of the ligand and Zn(II) complex.

Compound	^ 1^H NMR *δ* (ppm)	^ 13^C NMR *δ* (ppm)
Schiff base (L) C_23_H_20_S_2_O_8_N_5_Na	2.10 (3H, s, COOCH_3_); 4.72, 4.90 (2H, AB, *J* = 12.2 Hz, C_3_–CH_2_); 3.40, 3.67 (2H, AB, *J* = 17.2 Hz, C_2_–H_2_); 5.22 (1H, d, *J* = 4.6 Hz, C_5_–H *β*-lactam); 5.79 (1H, d, *J* = 4.6 Hz, C_7_–H *β*-lactam); 9.53 (1H, s, –NH–CO); 3.99 (3H, s, OCH_3_); 6.73 (1H, s, thiazolyl–C_16_–H); 8.7 (1H, s, –HC=N); 10.2 (1H, s, –OH); 7.5 (4H, m, –phenyl)	163.0; 143.4; 109.9 (C_17_; C_15_; C_16_–thiazole ring); 168.39 (C_13_); 169.3; (C_12_); 58.12; 65.4; 171.4 (C_5_; C_7_; C_6_ *β*-lactam); 139.5; 112.2; 26.2 (C_4_; C_3_; C_2_); 58.3 (C_9_); 163.9 (C_10_); 21.6 (C_11_); 165.0 (C_8_); 62.7 (C_14_); 163.62 (–HC=N); 157.82 (C_20_ phenyl); 132.24; 130.46; 121.25; 117.43; 112.87 (C_19_, C_21_, C_22_, C_23_, C_24_ phenyl)

ZnL_2_(H_2_O)_2_ ZnC_46_H_42_S_4_O_18_N_10_Na_2_	2.10 (3H, s, COOCH_3_); 4.72, 4.90 (2H, AB, *J* = 12.2 Hz, C_3_–CH_2_); 3.40, 3.67 (2H, AB, *J* = 17.2 Hz, C_2_–H_2_); 5.22 (1H, d, *J* = 4.6 Hz, C_5_–H *β*-lactam); 5.79 (1H, d, *J* = 4.6 Hz, C_7_–H *β*-lactam); 9.53 (1H, s, –NH–CO); 3.99 (3H, s, OCH_3_); 6.73 (1H, s, thiazolyl–C_16_–H); 8.96 (1H, s, –HC=N); 7.5 (4H, m, –phenyl)	163.0; 143.4; 109.9 (C_17_; C_15_; C_16_ thiazole ring); 168.39 (C_13_); 169.3; (C_12_); 58.12; 65.4; 171.4 (C_5_; C_7_; C_6_ *β*-lactam); 139.5; 112.2; 26.2 (C_4_; C_3_; C_2_); 58.3 (C_9_); 163.9 (C_10_); 21.6 (C_11_); 165.0 (C_8_); 62.7 (C_14_); 168.16 (–HC=N); 157.82 (C_20_ phenyl); 132.24; 130.46; 121.25; 117.43; 112.87 (C_19_, C_21_, C_22_, C_23_, C_24_ phenyl)

**Table 2:** Relevant IR data (cm^−1^) of the ligand and its complexes.

Compound	*ν*(OH)	*ν*(NH_2_)	*ν*(OH) associate	*ν*(C=O) *β*-lact	*ν*(C=N) azm.	*ν*(C=O) amide *ν*(C=O) ester	*ν*(C–O) phenolic	*δ*_*r*_(H_2_O) *δ*_*w*_(H_2_O) coord.	*ν*(M–O) *ν*(M–N)
Cefotaxime-Na	—	3442	—	1776	—	1640	—	—	—
Schiff base (L)	—	—	2800	1770	1657	1645	1274	—	—
[CoL_2_(H_2_O)_2_]	3530	—	—	1770	1624	1640	1295	863 539	419 510
[NiL_2_(H_2_O)_2_]	3545	—	—	1770	1623	1640	1305	863 541	420 520
[CuL_2_(H_2_O)_2_]	3540	—	—	1770	1620	1645	1295	860 545	427 514
[ZnL_2_(H_2_O)_2_]	3543	—	—	1770	1630	1640	1310	857 545	423 517

**Table 3:** Absorption maxima from electronic spectra, magnetic moments, and crystal field parameters for Schiff base (L) and its complexes.

Compound	Absorption maxima (cm^−1^)	Assignments	*µ*_eff_ (B.M.)	Crystal field parameters		
				10Dq (cm^1^)	*B* (cm^−1^)	*β*
L	38460 28570	*π* → π* *n* → π*	—	—	—	—
[CoL_2_(H_2_O)_2_]	22936 19305 (*ν*_3_) 9570 (*ν*_1_)	*n* → π* ^4^T_1g_(F) → ^4^T_1g_(P) ^4^T_1g_ → ^4^T_2g_(F)	4.78	1071	724	0.748
[CuL_2_(H_2_O)_2_]	23094 14290 (*ν*_1_)	*n* → π* *d*_*xy*_ → *d*_*x*^2^−*y*^2^_	1.86	1429	—	—
[NiL_2_(H_2_O)_2_]	27070 24880 (*ν*_3_) 16630 (*ν*_2_) 10120 (*ν*_1_)	*n* → π* ^3^A_2g_(F) → ^3^T_1g_(P) ^3^A_2g_(F) → ^3^T_1g_(F) ^3^A_2g_(F) → ^3^T_2g_(F)	3.12	1012	793	0.752
[ZnL_2_(H_2_O)_2_]	19800	C.T. (L → M)		—	—	—

**Table 4:** Thermogravimetric data of Co(II), Ni(II), and Cu(II) complexes.

Complex	Steps	Thermal effect	Temperature range (°C)	Δ*m*_exp⁡_ %	Δ*m*_calc_ %	Lost fragment
[CoL_2_(H_2_O)_2_] CoC_46_H_42_S_4_O_18_N_10_Na_2_	1	Endothermic	70–117	11.53	11.63	C_6_O_4_H_10_
	2	Exothermic	117–197	2.55	2.86	2*x*H_2_O
	3	Exothermic	197–294	32.71	32.67	C_14_H_8_O_6_S_2_N_2_Na_2_
	4	Endothermic	294–453	15.61	15.93	C_6_O_4_H_8_N_4_
	5	Exothermic	453–595	32.75	32.19	degradation products +
				4.58	4.69	metallic oxide residue

[NiL_2_(H_2_O)_2_] NiC_46_H_42_S_4_O_18_N_10_Na_2_	1	Endothermic	75–125	11.73	11.63	C_6_O_4_H_10_
	2	Exothermic	125–205	2.35	2.86	2*x*H_2_O
	3	Exothermic	205–302	31.71	32.67	C_14_H_8_O_6_S_2_N_2_Na_2_
	4	Endothermic	302–460	14.61	15.93	C_6_O_4_H_8_N_4_
	5	Exothermic	460–602	31.75	32.19	degradation products +
				4.78	4.72	metallic oxide residue

[CuL_2_(H_2_O)_2_] CuC_46_H_42_S_4_O_18_N_10_Na_2_	1	Endothermic	80–127	11.79	11.59	C_6_O_4_H_10_
	2	Exothermic	127–207	2.55	2.85	2*x*H_2_O
	3	Exothermic	207–304	32.71	32.55	C_14_H_8_O_6_S_2_N_2_Na_2_
	4	Endothermic	304–463	15.61	15.87	C_6_O_4_H_8_N_4_
	5	Exothermic	463–605	32.15	32.07	Degradation products +
				4.98	5.04	metallic oxide residue

**Table 5:** Structural descriptors of the studied compounds.

Compound	Interatomic distances, Å						
	C_37_–C_38_	C_38_–C_39_	N_1_–C_46_	N_1_–C_2_	C_2_–C_3_	C_3_–C_4_	C_7_–N_13_
L	1.4394	1.5182	1.4435	1.3865	1.5110	1.3466	1.3412
[NiL_2_(H_2_O)_2_]	1.4093	1.5297	1.4520	1.3728	1.3602	1.3596	1.2647
[CuL_2_(H_2_O)_2_]	1.4091	1.5298	1.4519	1.3727	1.3601	1.3569	1.2642
[ZnL_2_(H_2_O)_2_]	1.4092	1.5299	1.4518	1.3726	1.3600	1.3568	1.2634
[CoL_2_(H_2_O)_2_]	1.4092	1.5300	1.4519	1.3726	1.3601	1.3570	1.2643

**Table 6:** Structural descriptors of the studied compounds.

Compound	*E* (kcal/mol)	*M* (g/mol)	*μ* (D)	*α* (Å^3^)	*V* (Å^3^)	OV
L	80.277	559.57	6.415	53.32	1361.67	1.6856
[NiL_2_(H_2_O)_2_]	277.223	1211.86	11.26	107.54	2603.47	1.9871
[CuL_2_(H_2_O)_2_]	287.481	1216.70	11.55	107.58	2605.22	1.9814
[ZnL_2_(H_2_O)_2_]	284.497	1218.52	11.69	107.59	2613.30	1.9800
[CoL_2_(H_2_O)_2_]	285.611	1212.08	11.56	107.56	2604.48	1.9815

**Table 7:** Qualitative screening of the susceptibility spectra of various microbial strains to the synthesized compounds (mm).

Number	Compound	*E. coli *13147	*E. coli *13529	*K. pneumoniae *1204	*K. pneumoniae *13420	*P. aeruginosa *1246	*P. aeruginosa *13202	*B. subtilis *12488	*S. aureus* MRSA 1263	*S. aureus *13209
1	L	—	6	—	—	—	—	—	—	7
2	[NiL_2_(H_2_O)_2_]	—	6	7	—	—	—	—	8	11
3	[CuL_2_(H_2_O)_2_]	16	14	17	7	—	—	14	15	15
4	[ZnL_2_(H_2_O)_2_]	7	7	7	9	7	6	10	11	9
5	[CoL_2_(H_2_O)_2_]	11	16	8	6	6	8	7	8	12
DMSO-control		—	—	—	—	3	—	—	6	5
Antibiotic control	Cefotaxime	—	7	6	7	6	6	6	—	—

**Table 8:** Minimum inhibitory concentration (MIC) (µg/mL) of the synthesized compounds.

Number	Compound	*E. coli* 13147	*E. coli* 13529	*K. pneumoniae* 1204	*K. pneumoniae* 13420	*P. aeruginosa* 1246	*P. aeruginosa* 13202	*B. subtilis* 12488	*S. aureus* MRSA 1263	*S. aureus* 13209
1	L	—	0.5	—	—	—	—		—	—
2	[NiL_2_(H_2_O)_2_]	—	0.5	1	—	—	—	—	—	1
3	[CuL_2_(H_2_O)_2_]	0.0625	0.033	0.125	0.5	—	—	1	1	0.5
4	[ZnL_2_(H_2_O)_2_]	0.5	0.25	0.5	0.5	1	—	1	1	1
5	[CoL_2_(H_2_O)_2_]	1	0.25	1	0.5	—	1	1	1	1
DMSO-control		1	1	1	1	1	1	1	1	1
